# Finger Prick Dried Blood Spots for HIV Viral Load Measurement in Field Conditions in Zimbabwe

**DOI:** 10.1371/journal.pone.0126878

**Published:** 2015-05-22

**Authors:** Sue Napierala Mavedzenge, Calum Davey, Tarisai Chirenje, Phyllis Mushati, Sibongile Mtetwa, Jeffrey Dirawo, Boniface Mudenge, Andrew Phillips, Frances M. Cowan

**Affiliations:** 1 Women’s Global Health Imperative, RTI International, San Francisco, California, United States of America; 2 London School of Hygiene and Tropical Medicine, London, United Kingdom; 3 Centre for Sexual Health and HIV/AIDS Research Zimbabwe, Harare, Zimbabwe; 4 Flowcytometry Laboratory, Harare, Zimbabwe; 5 University College London, London, United Kingdom; University of Liverpool, UNITED KINGDOM

## Abstract

**Background:**

In the context of a community-randomized trial of antiretrovirals for HIV prevention and treatment among sex workers in Zimbabwe (the SAPPH-IRe trial), we will measure the proportion of women with HIV viral load (VL) above 1000 copies/mL (“VL>1000”) as our primary endpoint. We sought to characterize VL assay performance by comparing results from finger prick dried blood spots (DBS) collected in the field with plasma samples, to determine whether finger prick DBS is an acceptable sample for VL quantification in the setting.

**Methods:**

We collected whole blood from a finger prick onto filter paper and plasma samples using venipuncture from women in two communities. VL quantification was run on samples in parallel using NucliSENS EasyQ HIV-1 v2.0. Our trial outcome is the proportion of women with VL>1000, consistent with WHO guidelines relating to regimen switching. We therefore focused on this cut-off level for assessing sensitivity and specificity. Results were log transformed and the mean difference and standard deviation calculated, and correlation between VL quantification across sample types was evaluated.

**Results:**

A total of 149 HIV-positive women provided DBS and plasma samples; 56 (63%) reported being on antiretroviral therapy. VL ranged from undetectable-6.08 log_10_ using DBS and undetectable-6.40 log10 using plasma. The mean difference in VL (plasma-DBS) was 0.077 log_10_ (95%CI = 0.025–0.18 log_10_; standard deviation = 0.63 log_10_,). 78 (52%) DBS and 87 (58%) plasma samples had a VL>1000. Based on plasma ‘gold-standard’, DBS sensitivity for detection of VL>1000 was 87.4%, and specificity was 96.8%.

**Conclusion:**

There was generally good agreement between DBS and plasma VL for detection of VL>1000. Overall, finger prick DBS appeared to be an acceptable sample for classifying VL as above or below 1000 copies/mL using the NucliSENS assay.

## Background

Data suggest that female sex workers (FSW) in Zimbabwe have an HIV prevalence ranging from 50–70%, however they are poorly engaged in prevention and care services.[1] In response, we are implementing a community-randomized trial of antiretrovirals for HIV prevention and treatment among FSW in Zimbabwe—the SAPPH-IRe trial (PACTR201312000722390). The SAPPH-IRe trial is a matched-pair cluster-randomized trial conducted in 14 communities of FSW in Zimbabwe.[2] The primary objectives are i) to decrease the proportion of FSW with a viral load (VL) above 1000 copies/mL, due to VL suppression on ART or reduced new infections, by implementing an ‘enhanced intervention’ in addition to that provided by an existing network of sex worker clinics; and ii) to determine the likely effects of the enhanced intervention on the general population in Zimbabwe. As our primary endpoint in the SAPPH-IRe trial we will measure the proportion of FSW with a HIV VL>1000 copies/mL (“VL>1000”).

Implementation and scale-up of VL testing for the clinical management of ART is recommended by the World Health Organization (WHO).[3, 4] Routine VL monitoring is now the preferred approach for diagnosis and confirmation of ART failure. Despite this recommendation, VL measurement using plasma is logistically challenging and costly, and is therefore not routinely available in most resource-limited settings. Plasma sampling requires a phlebotomist for venipuncture, transportation to laboratory facilities for processing and analysis within 24 hours or availability of a reliable cold chain, and if required, long term storage at -20°C. Collecting dried blood spots (DBS) using a finger prick blood sample, by comparison, does not require venipuncture, samples are prepared on collection cards that can be stored at ambient tropical temperatures for weeks without RNA degradation, thus facilitating transportation and storage from rural areas to a central laboratory.[5] This greatly reduces both the resources required and logistical challenges of VL testing.

Recent studies have demonstrated good correlation between DBS and plasma samples in resource-limited settings including Malawi, Kenya, Nigeria and Senegal using various platforms.[6] However few studies have evaluated the performance of DBS using a finger prick sample (rather than created from a venipuncture sample in a laboratory),[7–9] which may impact VL results due to difficulty with standardizing the volume of whole blood collected. Furthermore, few data exist from samples collected under field conditions in Africa.[10] Further research is therefore required to determine whether a whole blood sample collected by finger prick for DBS is an acceptable sample for plasma VL quantification, and whether it can be successfully collected and analyzed in the context of a field study. In this study we sought to characterize VL assay performance under field conditions using the NuclisENS EasyQ HIV-1 v2.0 assay with finger prick DBS as compared with plasma samples, to determine whether DBS is an acceptable sample for VL quantification in our setting.

## Methods

In the context of the SAPPH-IRe trial baseline survey, we recruited FSW between November and December 2014 for this validation study from two of 14 SAPPH-IRe communities in Zimbabwe using respondent driven sampling. Participants had to be >18 years of age, currently working as a sex worker (defined as having exchanged sex for money in the past 30 days) and able to provide informed consent in order to be eligible to participate.

Ethical approval was obtained from the Medical Research Council of Zimbabwe, as well as the University College London, London School of Hygiene and Tropical Medicine, and RTI International (formerly Research Triangle Institute) institutional review boards (IRB) prior to initiating study procedures. All participants provided written informed consent which was recorded on the consent form. All IRBs approved this consent procedure.

A standardized questionnaire was administered to collect data on demographics and sexual behaviour, as well as on HIV testing history, HIV status, engagement in HIV prevention and care services and use of antiretroviral therapy (ART). We collected both finger prick DBS and plasma samples from each participant in parallel. Five finger prick samples per participant were collected directly onto Whatman 903 blood spot collection cards by a nurse. Samples are collected onto circles on the collection cards. A punch is taken from within each DBS circle, which contains a fixed volume of blood equaling 0.050 mL per punch (or 50 μL). DBS samples were dried overnight, placed in gas impermeable ziplock bags and stored at room temperature with desiccants for transportation to the laboratory for testing. Whole blood was drawn into a BD Vacutainer EDTA tube to prepare plasma samples. The EDTA blood was centrifuged at 400G for 5 minutes and 0.5mL plasma was transferred to the Nuclisense Lysis buffer tubes and vortexed thoroughly within 24 hours of collection. The plasma was then stored between 2–8°C until testing within 14 days.

All DBS samples were tested for HIV antibody using AniLab ELISA (Anilab Systems, Finland). For those samples which were positive for HIV, VL quantification was run on both plasma and DBS samples in parallel. RNA isolation from DBS was performed by punching off two 50uL blood spots into a 2mL tube of Nuclisens Lysis buffer and incubated on a roller mixer for 30 minutes. The tubes were then centrifuged for 15 seconds at 1500G. Of this lysed specimen, 1.8mL was transferred for nucleic acid extraction. VL testing for both DBS and plasma samples was carried out using the NucliSENS EasyQ HIV-1 v2.0 assay (bioMerieux) following manufacturer’s instructions. The manufacturer specified lower limit of quantification for 0.1mL DBS was 802 copies/mL and for 0.5mL plasma was 50 copies/mL. The laboratory was able to achieve a lower limit of quantification of 100 copies/mL for DBS and 20 copies/mL for plasma. For the purposes of this study, the primary endpoint is a VL>1000 copies/mL, consistent with WHO guidelines relating to regimen switching.[3]

### Statistical methods

VL results for both DBS and plasma samples were log transformed and expressed as means and standard deviations. VL measured on plasma served as the reference for all comparison calculations. We described the VL range, proportion with VL>1000 for each sample type, and mean difference (plasma-DBS). Specimens were divided into strata by plasma VL and compared to paired DBS samples. Sensitivity and specificity of DBS VL for identification of VL>1000 were calculated against plasma VL. Scatter plots were used to evaluate correlation between DBS and plasma VL, and a Bland-Altman plot was used to assess agreement between DBS versus plasma VL. All analyses were conducted using STATA 13 (StataCorp LP, Texas, USA).

## Results

A total of 149 FSW from the two communities tested positive for HIV and provided both DBS and plasma samples. Of the 149 HIV-infected women in our sample, 89 (60%) reported that they had been diagnosed with HIV, and 56 (38%) reported being on ART.

Quantitative VL using DBS samples ranged from undetectable-6.08 log_10_ and ranged from undetectable-6.40 log_10_ using plasma. Seventy eight (52%) of DBS and 87 (58%) of plasma samples had a VL>1000. Specimens were classified into strata by plasma VL (Table 1). Among samples with a plasma VL in the 1000–2999 copies/mL stratum, 31% fell in this range using DBS, while 100% and 97% fell in the 3000–4999 copies/mL and ≥5000 copies/mL strata, respectively.

**Table 1:** Plasma viral load and corresponding HIV RNA detection rates in dried blood spots.

Plasma viral load	Number of samples	VL > 1000 in DBS	VL > 1000 proportion % (95% CI)
<1000 copies/mL	62	2	3.2 (0.4–11.2
1000–2999 copies/mL	13	4	30.8 (9.1–61.4)
3000–4999 copies/mL	8	8	100 (63.1–100)
≥5000 copies/mL	66	64	97.0 (89.5–99.6)

The mean difference, or bias, in VL (plasma-DBS) was 0.077 log_10_ (standard deviation = 0.63 log_10_). VL values between DBS and plasma differed by less than 0.5 log_10_ in 108/149 (72%) of pairs. The sensitivity of DBS was 87.4% (78.4–92.9%) and the specificity was 96.8% (87.6–99.2%) against the plasma VL “gold standard” above the VL threshold of 1000 copies/mL (Table 2). Among those on ART (n = 56), sensitivity was 91.7% (49.9–99.2%) and specificity was 97.7% (84.5–99.7%). Among those who knew they were HIV-infected and reported not being on ART (n = 32) sensitivity was 86.5% (76.4–92.7%) and specificity was 94.4% (64.5–99.4%; data not shown).

**Table 2:** Qualitative agreement between plasma and DBS viral load.

	DBS			
Plasma	Number VL>1000	Number VL<1000	Total	
**Number VL>1000**	76	11	87	**Sensitivity: 87.4% (78.4–92.9%)**
**Number VL<1000**	2	60	62	**Specificity: 96.8% (87.6–99.2%)**
**Total**	78	71	149	
	**PPV: 97.4% (90.0–99.4%)**	**NPV: 84.5% (73.8–91.3%)**		

PPV: positive predictive value.

NPV: negative predictive value.

**Fig 1:**
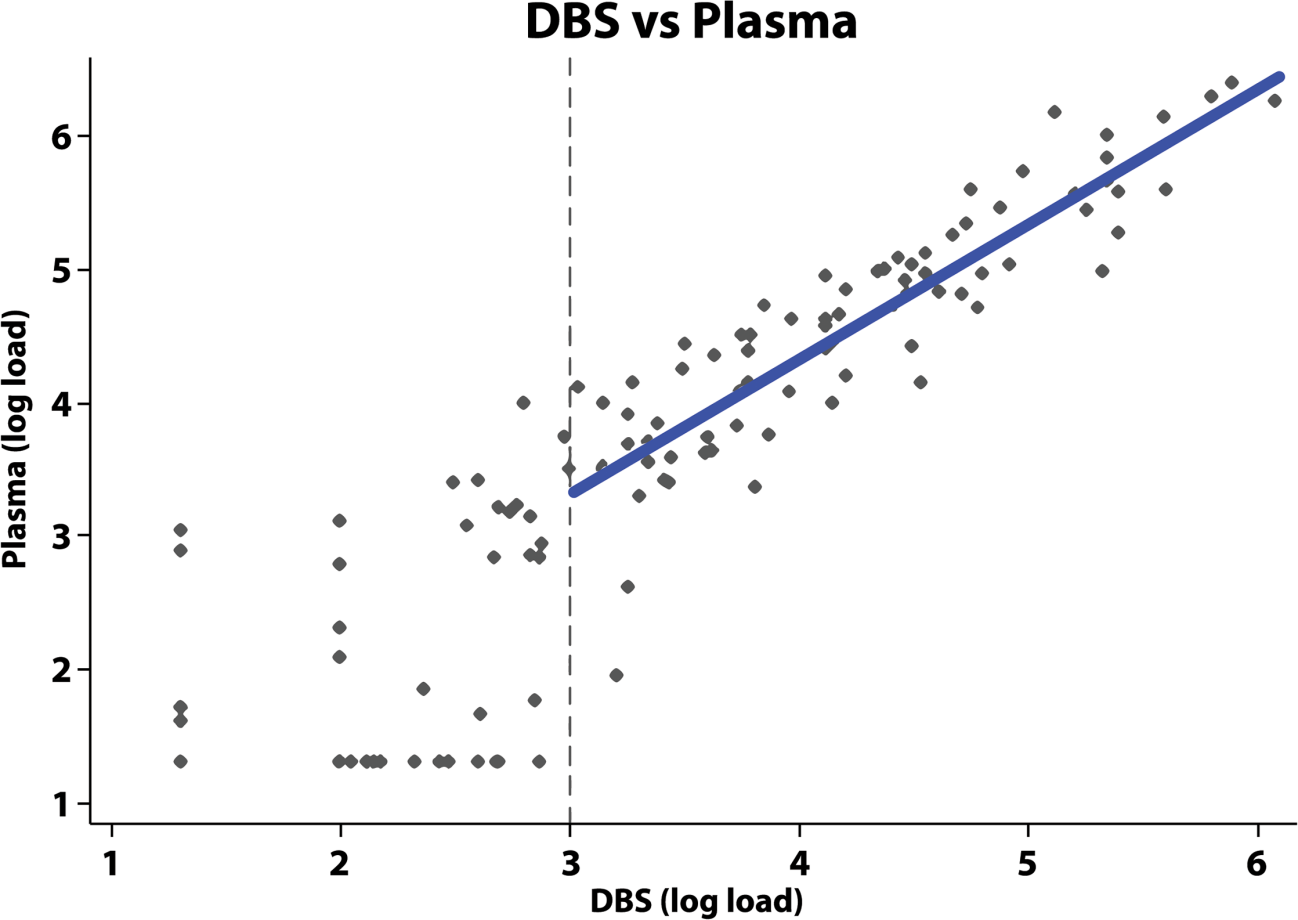
Correlation between measurement of DBS versus plasma viral load. The vertical dotted line represents the cut-off for WHO guidelines relating to regimen switching of 3 log_10_. Where the DBS sample load was above 1000 copies, a line was fitted with ordinary-least-squares regression to the log of the viral load results from the DBS and plasma samples. The resulting line was: log_10_(Load_*Plasma*_) = 0.29 + 1.01 * log_10_(Load_*DBS*_).

**Fig 2:**
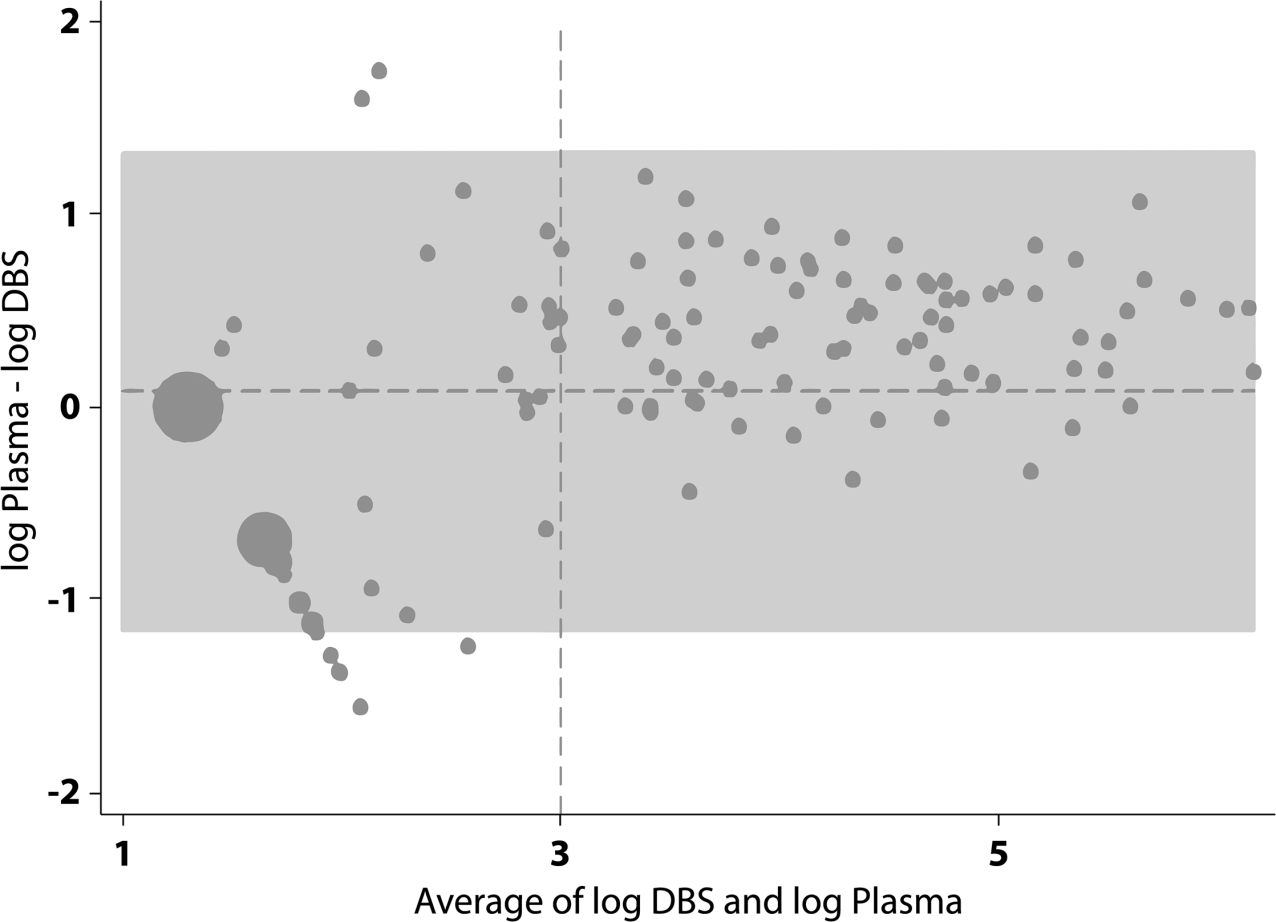
Bland-Altman plot of the agreement between DBS versus plasma viral load. The horizontal dotted line represents the mean difference of 0.077 log_10_. The grey area represents ±1.96 standard deviations. The vertical dotted line represents the 3 log_10_ cut-off for detectable viral load.

Fig 1 shows the correlation between DBS and plasma samples above the threshold for a detectable VL of 3 log_10_ (linear coefficient = 1.0, 95%CI = 0.90–1.13; R-squared = 0.79). A Bland-Altman plot of the agreement between DBS versus plasma VL indicated good agreement above 3 log_10_ (VL>1000), with all paired samples falling within +/- 1.2 logs (Fig 2).

## Discussion

Evaluating the performance of finger prick DBS as a more logistically feasible and cost-saving sample for plasma VL quantification is a high priority in resource-limited settings. The performance of DBS samples has been evaluated in other studies and been shown to have good sensitivity and specificity.[6] However few data are available from resource-limited settings,[11–16] and finger prick DBS samples (rather than those created from a venipuncture sample) have rarely been evaluated.[7–9] To our knowledge this is the first study to evaluate the feasibility of using finger prick DBS samples for the measurement of plasma VL as a primary research endpoint, and one of the few to collect finger prick DBS samples under field conditions in sub-Saharan Africa.[10]

We found excellent correlation and good agreement between finger prick DBS and the plasma VL “gold standard” above the 3 log_10_ (VL>1000) cut-off for viral suppression using the NucliSENS v2.0 assay. In addition, we found good sensitivity and specificity for VL >1000, similar to that found in other studies of DBS prepared from venipuncture sample using the same assay.[4] A small proportion of women may be classified as VL<1000 using DBS when the VL is >1000 based on a plasma sample. However, quantification in plasma itself is also associated with some within-sample assay variability.[17]

The WHO now recommends using the cut-off of 1000 copies/mL (i.e. 3 log_10_) to define virologic failure, rather than the previous guidelines of 5000 copies/mL.[3] While detection using DBS samples was 31% in paired samples in the range of 1000–2999 copies/mL ^3^, detection in paired samples with a VL over 3000 copies/mL was near perfect. Though these results indicate room for improvement in terms of the sensitivity of finger prick DBS at lower VL, for the purposes of measuring population level virological suppression in the context of our research, finger prick DBS is an adequate specimen type. In terms of clinical management of HIV infection, the lower sensitivity represents a disadvantage because some people with VL 1000–2999 copies/mL will be misclassified. However, the lack of evidence for what is the most appropriate cut-off, balancing reduction in resistance accumulation and risk of transmission on the one hand with the risk of unnecessary early switch to a more expensive second line regimen on the other, means that the consequences of misclassifying some people with plasma VL 1000–2999 as <1000 is of unclear practical concern. DBS is, and may always be less sensitive when compared to plasma for samples with low VL levels, due primarily to the smaller sample volume. However it may still be a reasonable option for clinical management of HIV and identification of treatment failure in contexts where taking a venipuncture sample and ensuring its correct handling and processing may not be feasible, particularly when there is longitudinal follow up of individuals. Additionally, if the only realistic alternative for clinical management is CD4 count monitoring then the small loss of accuracy compared with plasma may well be acceptable, as viral load measured on a DBS sample is likely to be a better indicator of whether virologic failure has occurred than is the CD4 count. Modelling studies can help us understand the net balance of these trade-offs.

There were a number of strengths in the methodology employed in this research. We used the Whatman 903 collection cards for DBS sampling. This is one of the collection cards recommended for use by the WHO in terms of having sufficient evidence of performance.[4] We processed our samples within 24 hours of collection and tested all samples with 14 days. Samples were run in parallel using similar VL quantification methodology after sample extraction. We used the NucliSENS EasyQ HIV-1 v2.0 assay, which is among the assays for which the WHO has provided provisional data on performance characteristics for use with DBS.[4] The NucliSENS assay also had the advantage that it amplifies only plasma and not cellular DNA. There is potential for variability in sample collection across field staff and study setting in this research, which could have impacted results of VL quantification. This issue, however, is one that is routinely encountered in the context of program implementation and scale-up.

Despite WHO’s recommendation to implement and scale up the use of VL testing for clinical management of ART, VL technology remains out of reach for routine monitoring in most resource-limited settings. There is a need to facilitate access to VL measurement, for the purposes of research as well as clinical management. The high cost and stringent requirements for storage and transport of plasma make finger prick DBS an ideal alternative, as a technologically simpler, less invasive and less expensive method of measuring VL. Point-of-care technologies are also being developed for VL measurement, so there may soon be an additional alternative for VL monitoring. In the context of the SAPHH-IRe trial, we have determined that the sensitivity and specificity achieved using finger prick DBS as compared to plasma sample, and the correlation between samples, is adequate for research purposes and will be used in our repeat surveys to ascertain the proportion of women with suppressed VL at the end of the SAPPH-IRe intervention. Results from this research additionally add to the body of knowledge on the use of finger prick DBS as an alternative sample for VL measurement in the clinical management of ART in resource-limited settings.
